# The relationship between childhood trauma and depressive symptom among Zhuang adolescents: Mediating and moderating effects of cognitive emotion regulation strategies

**DOI:** 10.3389/fpsyt.2022.994065

**Published:** 2022-09-06

**Authors:** Wenwen Yin, Yuli Pan, Linhua Zhou, Qiaoyue Wei, Shengjie Zhang, Hong Hu, Qinghong Lin, Shuibo Pan, Chenyangzi Dai, Junduan Wu

**Affiliations:** ^1^Department of Psychology, School of Public Health, Guangxi Medical University, Nanning, China; ^2^Department of Guangxi Zhuang Autonomous Region Center for Disease Control and Prevention, Nanning, China; ^3^Department of Graduate Management, Guangxi Medical University Cancer Hospital, Nanning, China; ^4^Department of Medical Institution, Guangxi Nanning Fifth People's Hospital, Nanning, China; ^5^Department of School of Public Health, Guangxi Medical College, Nanning, China

**Keywords:** childhood trauma, depression, expressive suppression, the Zhuang adolescents, emotional abuse in childhood

## Abstract

**Background:**

Not all adolescents who have endured childhood trauma will develop depressive symptom, nor will they all experience the same level of depressive symptom. According to previous research, cognitive emotion regulation strategies may explain a portion of the variance. Observe the connection between childhood trauma and depressive symptom and investigate whether cognitive emotion regulation strategies mediate or moderate this association.

**Methods:**

In October 2019, a cross-sectional study measuring childhood trauma, cognitive emotion regulation strategies, and depressive symptom among Zhuang adolescents was done in one senior high school and two junior highs in Chongzuo, Guangxi, China, using a self-report questionnaire. To examine the hypothesis of mediating and moderating effects, SPSS PROCESS was utilized.

**Results:**

In this study, there was a positive relationship between childhood trauma and depressive symptom, whereas there were positive correlations between expressive suppression and childhood trauma and depressive symptom (*r* = 0.380, 0.246, and 0.089, respectively, *p* < 0.01). The 5,000-sample bootstrap procedure revealed that the indirect relationship between the independent variable (childhood trauma or emotional abuse) and the dependent variable (depressive symptom) was statistically significant (β = 0.0154 95% CI: 0.0019, 0.0165, β = 0.0442 95% CI: 0.0008, 0.0117). The statistical significance of the interaction effect enhanced the R-square value of the moderating effect when the independent variable was the total childhood trauma score (ΔR2 = 0.0044, 0.0089).

**Conclusions:**

Our findings corroborated the conclusion of prior research that cognitive emotion regulation strategies mediate and moderate the development of depressive symptom. Although we demonstrate that cognitive emotion regulation strategies play a mediating and moderating role in the relationships between childhood trauma and depressive symptom, the mediating effects on the relationships between the other types of childhood traumas, including physical abuse and neglect, sexual abuse, emotional neglect, and depressive symptom, did not emerge.

## Introduction

According to the World Health Organization (WHO), over a third of the global population has suffered childhood trauma. An estimated one-fifth of women and one-thirteenth of males have experienced sexual abuse in childhood, while around one-quarter of adults have experienced physical abuse in childhood (1). Additionally, 28.9 % of individuals with mental disorders reported having experienced childhood trauma, and the repercussions of early trauma may last a lifetime. As demonstrated by numerous earlier studies, childhood trauma was considered a particularly substantial risk factor for the onset, symptomatology, and course of depressive symptom (2, 3). As a major psychiatric disorder, depression poses a formidable public health concern. Around the world, 264 million people of all ages suffered from depression (4), with adolescents being particularly at risk (5). As a subclinical stage of depression, depressive symptom has garnered great attention due to their ubiquity. According to the Global School-based Student Health Survey (GSHS), 28.7 % of 67,077 teenagers exhibited depression symptom (6). Multiple areas' research conducted in China revealed that 20.3% of adolescents experienced depressive symptom (7). Therefore, in order to investigate the pathogenesis, prevention, and treatment of adolescent depression, it is vitally important to examine depressive symptom. A significant correlation exists between childhood trauma and adolescent depressive symptom, and adolescents who have suffered childhood trauma are more likely to develop depressive symptom than those who have not (8). The concept that childhood trauma is connected with the emergence and recurrence of depressive symptom is supported by abundant research (9). The study of the genesis of depressive symptom revealed that persons who suffer negative life occurrences are susceptible to depressive symptom (10). When confronted with the same condition, however, individuals react differently: some suffer depressive symptom, while others do not. This phenomenon enlightens individuals about the fact that certain variables determine stress situations that lead to depressive symptom. According to studies, depressive symptom may develop due to acute or chronic life stress, particularly in individuals who have undergone childhood trauma (11).

However, Insufficient research has been conducted on the causative mechanisms between childhood trauma and the eventual development of depressive symptom. In a large number of extant studies on childhood trauma and depressive symptom, the variables that influence the link between childhood trauma and depressive symptom can be categorized into two major groups: psychosocial and neurobiological. Psychosocial aspects include cognitive elements (12, 13), emotion regulation components (14) and personality factors (15). Furthermore, diverse putative mediators of the association between childhood trauma and depressive symptom have been identified, but elucidating research remains scant. A mediator variable in a mediation model can be used to further explain the relationship between a predictor variable and an outcome variable (16). The literature has so far identified a number of putative mediators of the link between childhood trauma as a youngster and depressive symptom. In a nationally representative epidemiological investigation, self-criticism was discovered to be a mediator of the relationship between parental verbal abuse and internalizing symptom (depressive symptom and anxiety) in adulthood (17). Additionally, it has been discovered that patterns of vulnerability to harm, shame, and self-sacrifice modulate the impact of emotional abuse on anxiety and depressive symptom that develop later in teenagers (18).

The inadequate development of effective cognitive emotion control techniques after childhood maltreatment may be another important factor linking childhood trauma to depression in adults (19). Cognitive emotion regulation strategies are the cognitive response of an individual attempting to control the quantity and kind of their own emotional experience and the event itself in response to an emotion-eliciting stimulus (20). Gross and John identified cognitive reappraisal and expressive suppression as the two fundamental mechanisms for emotion regulation (21). Furthermore, cognitive reappraisal is an antecedent-focused strategy that refers to analyzing emotionally arousing situations to modify their emotional impact, whereas expression suppression is a response-oriented strategy that involves suppressing an individual's outward displays of emotional state. Cognitive reappraisal was associated with better emotions and enhanced functioning, whereas expressive suppression was linked to negative emotions and decreased functioning. A meta-analysis of emotion regulation strategies reveals that emotion-related disorders are more strongly associated with emotion regulation strategies than other problems, and maladaptive strategies are more consistently associated with psychopathology than adaptive ones (22). Furthermore, psychological issues have been demonstrated to be closely connected with emotion regulation strategies (23). In addition, early negative experiences may contribute to the creation of latent sad cognitive schema, which influences the emotions, behaviors, and ideas of depressive symptom patients (24). For example, physical abuse during childhood is associated with schemas of danger and mistrust, while emotional neglect is associated with schemas of value and belonging (25, 26). Individuals who experience childhood trauma usually struggle to interpret their feelings due to a lack of sufficient environmental feedback. Therefore, they employ avoidance and reflection as coping mechanisms, which might easily result in negative adaptation (27). The ability to use emotion regulation strategies is developed early in life, and some studies suggest that exposure to maltreatment as a youngster may have a negative impact on that child's subsequent ability to use emotion regulation strategies (28, 29). Additionally, it has been demonstrated that a number of mental health problems are linked to deficiencies in emotion regulation (30, 31). In summary, traumas in childhood may impact depressive symptom through cognitive emotion regulation strategies. Existing research has also investigated the role of cognitive emotion regulation as a mediator between later mental health disorders and childhood trauma (32, 33).

In this study, the Childhood Trauma Questionnaire-Short Form (CTQ-SF), the Emotional Regulation Questionnaire (ERQ), and the Patient Health Questionnaire-9 (PHQ-9) were used to examine the childhood trauma, cognitive emotion regulation mechanisms, and depressive symptom of teenagers individually. Compared to earlier investigation on mediation analysis of cognitive emotion regulation strategies, in which they are derived for these two regulation strategies (34), We measured the Zhuang adolescents' experiences of childhood trauma using the widely established CTQ-SF scale; another research utilized the CTQ scale but evaluated it exclusively from the standpoint of emotional and physical abuse (35). Moreover, little research has sought to simultaneously analyze cognitive emotion regulation strategies' mediating and moderating effects on the relationship between depressive symptom and childhood trauma in Chinese Zhuang adolescents. However, there is a paucity of comparative studies on Chinese ethnic minorities. Previous research has shown that young Zhuang people, as an ethnic minority, encounter greater obstacles than their Chinese peers due to their different language and lower levels of education, income, and professional opportunities (36, 37). These challenges may place Zhuang children at a substantial mental health disadvantage (38). In summary, this study used Zhuang adolescents as participants to analyze the connection between childhood trauma and depressive symptom and to determine whether cognitive emotion regulation strategies mediate and moderate this connection.

## Measurements and methods

### Participants

In October 2019, a cross-sectional study was undertaken using stratified cluster sampling at three schools (one senior high school and two junior highs) in Chongzuo, Guangxi, China. First, we randomly chose three campuses in Chongzuo, Guangxi. Second, ten classes were selected at random from the class roster. Thirdly, we invited all middle youngsters to complete questionnaire and collected them throughout the class. The questionnaire has a 60-min deadline for completion. Before providing informed consent and completing the questionnaire, all participants were informed about potential hazards and the complete confidentiality of information. Then, 1,494 questionnaires were returned, excluding those who declined to participate in the research. Due to insufficient data and logical errors in the survey, 362 questionnaires were excluded from the final analysis, leaving 1,132 questionnaires for analysis. Participants needed to be between 12 and 18 years old and of Zhuang descent to be included in the study. Additionally, the questionnaire was input using a response sheet and machine-readable card, which is accurate and efficient. The Institutional Ethical Committee authorized the research conducted at Guangxi Medical University (Approval Number: 20160302-13).

### Measurements

#### Participants' general information

Participants' basic demographic information was gathered, including age, gender, parental marital status, place of residence, resident student, only child, parental absence, teacher criticism, and study stress.

#### Childhood trauma

The Childhood Trauma Questionnaire Short-Form (CTQ-SF) was utilized to evaluate childhood trauma (39), a 28-item self-report questionnaire examining five varieties of traumas a child or adolescent has experienced: emotional and physical neglect; emotional, physical and sexual abuse. A 5-point frequency scale is used to grade items (1 = never true to 5 = very often true), with the total score for each type of trauma ranging from 5 to 25, with higher scores indicating more severity. Additionally, the Chinese version of the CTQ-SF has been confirmed (40). Moreover, in this study, Cronbach's alpha coefficient for the CTQ-SF was 0.737.

#### Emotional regulation questionnaire

The ERQ is a 10-item self-report instrument based on the process model of emotion regulation created by Gross (1998) (41). This model categorizes emotion regulation strategies based on how early they are triggered in the process of emotion creation and hypothesizes that different regulation strategies may have distinct outcomes. The ERQ is designed to examine the use of two regulation mechanisms by individuals: an antecedent-focused strategy called cognitive reappraisal (six items; e.g., “When I am faced with a stressful situation, I make myself think about it in a way that helps me stay calm”) where a person attempts to alter his or her perception of a circumstance in order to alter its emotional impact, and a response-focused strategy called expressive suppression (four items; e.g., “I keep my emotions to myself”) where an individual attempts to suppress the behavioral expression of their feelings (21). These two regulatory strategies are assigned distinct scale scores. Each item is graded on a 7-point Likert scale ranging from 1 (strongly disagree) to 7 (strongly agree), with higher scores indicating greater utilization of the method. In addition, Cronbach's alpha coefficient for the ERQ was 0.845.

#### Patient health questionnaire-9 items

This study utilized the patient health questionnaire-9 (PHQ-9) (42). The PHQ-9 was designed using the DSM-IV diagnostic criteria for depressive symptom. Participants evaluated their level of irritation with each item on a four-point Likert scale ranging from 0 (“not at all”) to 3. (“nearly every day”). Our recall period was 2 weeks. The overall score ranged from 0 to 27, with higher scores indicating greater depressive symptom severity as indicated by the individual. With a sensitivity of 80% and specificity of 92%, a total score of ≥ 10 predicted the possibility of severe depressive symptom (43, 44). Previously, psychometric characteristics of the PHQ-9 were validated in the Chinese population (42). In addition, Cronbach's alpha coefficient for the PHQ-9 was 0.878.

### Statistical analysis

The variables were subjected to descriptive and correlational statistical analyses using IBM SPSS version 25. The SPSS PROCESS macro was employed to examine the mediating and moderating effects hypotheses following Hayes' recommendations. The bootstrap procedure, thought to be the most precise by Hayes et al. was used to estimate the mediating effect of expressive suppression. The independent variable's direct impact on the mediator, including three direct effects and its total effect, the independent variable's direct effect on the dependent variable, the mediator's direct effect on the dependent variable, and the total effect of the independent variable on the dependent variable was evaluated, automatically. Whether the independent variable has a considerable impact on the dependent variable when the mediating effect has a significant impact determines whether there has been full or partial mediation. The moderator effect, the moderator's influence on the dependent variable, and the interaction effect on the dependent variable must all be statistically significant to test for a moderating effect. The statistical test was significant at the level of *P* < 0.05, according to the significance level.

## Results

### Participants' characteristics

The average age of 1,132 middle school students in this sample was 14.66 ± 1.441; 49.4% of the students were males, 73.4% were rural residents, and 12.6% were in the unstable marital status of parents, 21.6 % were the only child in their families, and 36.7% were parental absence. 9.3% of the students were never criticized by teacher, and most adolescents had the frequency of “teacher's criticism” sometimes. Furthermore, 91.3% of the students had study stress. The mean score of types of childhood trauma was 5.48–10.97. The mean score of CTQ-SF (Childhood trauma) was 28.4 ± 9.2; the expressive suppression score (Cognitive emotion regulation strategy), cognitive reappraisal score (Cognitive emotion regulation strategy), and the PHQ-9 score (Depressive symptom) is also shown in Table 1.

**Table 1:** Correlations of variable.

	**Mean (SD)**	**SA**	**EA**	**PA**	**EN**	**PN**	**CR**	**ES**	**CTQ-SF**	**DS**
SA	5.48 (1.305)	1.00								
EA	7.31 (2.848)	0.203**	1.00							
PA	5.91 (1.856)	0.182**	0.0380**	1.00						
EN	10.97 (5.154)	0.037	0.388**	0.185**	1.00					
PN	8.97 (3.406)	0.88**	0.294**	0.181**	0.541**	1.00				
CR	26.80 (8.399)	−0.03	−0.061**	−0.045	−0.239**	−0.206**	1.00			
ES	15.34 (5.614)	0.041	0.148**	0.049	0.037	0.043	0.404**	1.00		
CTQ-SF	38.65 (10.057)	0.270**	0.678**	0.472**	0.845**	0.744**	−2.19**	0.089**	1.00	
DS	7.17 (5.576)	0.108**	0.466**	0.154**	0.272**	0.192**	−0.07	0.246**	0.380**	1.00

^**^Correlation is significant at the 0.01 level (2-tailed). ES, expressive suppression; CR, cognitive reappraisal; SA, sexual abuse; EA, emotional abuse; PA, physical abuse; EN, emotional neglect; PN, Physical neglect; DS, depressive symptom.

### Correlation

Child trauma, the types of childhood trauma, emotion regulation (including expressive suppression and cognitive reappraisal), and depressive symptom were subjected to a bivariate Pearson correlation analysis; the correlation coefficients are shown in Table 1 below. Depressive symptom were favorably connected with childhood trauma, while expressive inhibition was positively associated with depressive symptom and childhood trauma (*r* = 0.380, 0.246, and 0.089, respectively, *p* < 0.01). Each type of childhood trauma had a positive connection with depressive symptom. Moreover, emotional abuse, a type of childhood trauma, was also positively related to depressive symptom, whereas expressive suppression was positively connected with depressive symptom and emotional abuse (*r* = 0.466, 0.246, and 0.148, respectively, *p* < 0.01). Additionally, cognitive reappraisal was negatively connected with childhood trauma, emotional abuse, and it was positively associated with expressive suppression (*r* = −2.19, −0.061 and 0.404, respectively, *p* < 0.01). The severity of childhood trauma correlates with the prevalence of expressive suppression and the risk of depressive symptom. Additionally, the same collection exists in emotional abuse. Consequently, the relation between cognitive reappraisal and depressive symptom had not statistically significant. (*r* = −0.07, *p* > 0.01), it was not included in the subsequent models. Furthermore, the relationship between other various childhood trauma, except for emotional abuse and expressive suppression, was not statistically significant. Hence, they were not included in the subsequent models.

### Mediating and moderating effects of expressive suppression

The correlations among the three variables were statistically significant and satisfied the requirements for examining the mediating and moderating effects. The score on childhood trauma or emotional abuse was the independent variable, the score on depressive symptom was the dependent variable, and the score on expressive suppression was the mediator or moderator variable; The scores were input into model 1 or 4 of SPSS PROCESS, and the variables age, gender, marital status of parents, place of residence, resident student, only child, parental absence, teachers' criticism, study stress was input as covariates. The results of the mediating effect are shown in Table 2. The statistical significance of each impact demonstrated the existence of a mediating influence.

**Table 2:** Result from PROCESS macro testing expressive suppression mediation model 1 and model 2.

**Effect^a^, Variable model 1**	**R^2^**	**F**	**β**	**P**
1.Direct effect of independent^b^ on mediator^c^	0.019	2.1693	0.808	0.0175
2.Direct effect of independent^b^ on dependent^d^	0.2768	38.9771	0.3212	<0.001
3.Direct effect of mediator^c^ on dependent^d^	0.2768	38.9771	0.1907	<0.001
4.Total effect of independent^b^ on dependent^d^	0.2412	35.6282	0.3366	<0.001
	β	95%CI		p
5.Indirect effect of independent^b^ on dependent ^d^	0.0154	0.0019–0.0165		<0.001
**Effect** ^**a**^ **, Variable model 2**	**R** ^**2**^	**F**	**β**	**P**
1.Direct effect of independent^e^ on mediator ^c^	0,300	3.4683	0.1357	0.0002
2.Direct effect of independent^e^ on dependent ^d^	0.3154	46.8736	0.3797	<0.001
3.Direct effect of mediator^c^ on dependent^d^	0.3154	46.8736	0.1665	<0.001
4.Total effect of independent^e^ on dependent^d^	0.2885	45.4206	0.4023	<0.001
	β	95%CI		p
5.Indirect effect of independent^e^ on dependent^d^	0.0442	0.0213, 0.0717		<0.001

^a^Adjusted for age, gender, place of residence, resident student, parental absence, marital status of parents, only-child, teacher criticism, study stress.

^b^Childhood trauma.

^c^Expressive suppression.

^d^Depressive symptoms.

^e^Emotional abuse.

**Figure 1:**
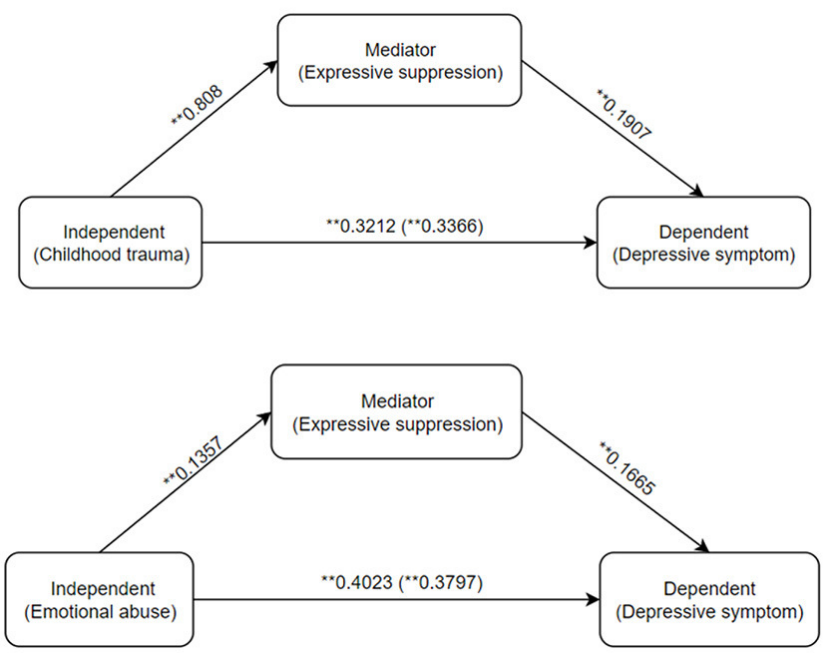
Output model of expressive suppression mediation. The ** symbol indicates the mediating effect is significant at the 0.01 level (2-tailed).

The bootstrap method with 5,000 samples revealed that the indirect influence of the independent variable (childhood trauma or emotional abuse) on the dependent variable (depressive symptom) was statistically significant (β = 0.0154 95% CI: 0.0019, 0.0165, β = 0.0442 95% CI: 0.0008, 0.0117). The ratio of the effect of mediation to the total effect was 45.75, or 10.98%. Figure 1 illustrates the mediating impact of expressive suppression. Childhood trauma increased the expressive suppression score, which in turn increased the depressive symptom score, hence increasing the risk of depressive symptom in adolescents. Emotional abuse has the same mechanism as Childhood trauma.

**Figure 2:**
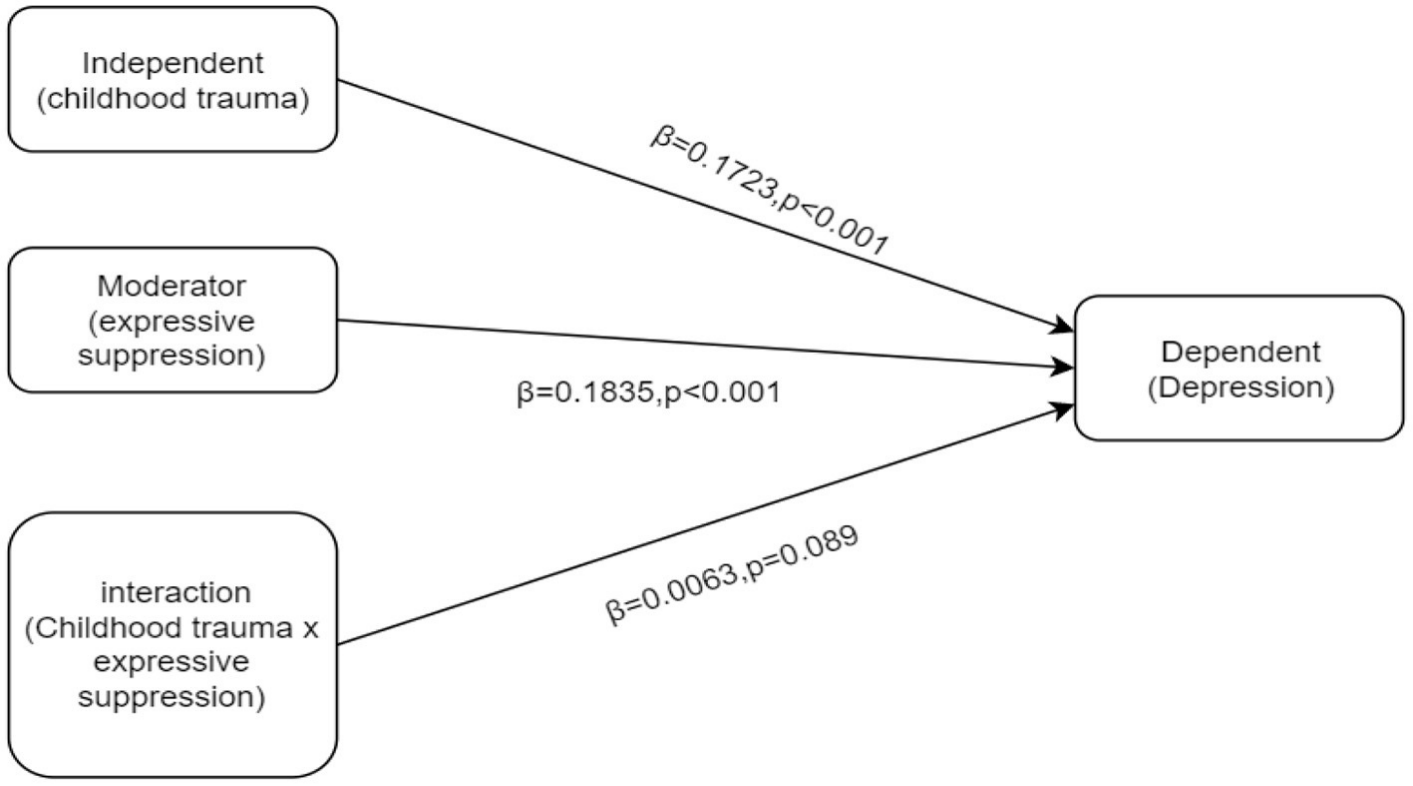
Output model of expressive suppression moderation.

Table 3 show the results of the moderating effect. In Table 3, the R2 increased due to the statistical significance of the interaction effect (ΔR2 = 0.0044, *p*= 0.0089). The bootstrap analyses of 5,000 samples revealed that the interaction effect was likewise statistically significant (β = 0.063, 95% CI: 0.0008,0.0117). However, in Table 3, the interaction effect was not statistically significant, according to the 5,000-sample bootstrap procedure (β = 0.0011, 95% CI: −0.0071, 0.0311). With growth of one standard deviation in expressive suppression, the childhood trauma to depressive symptom slope increased by 0.0063 standard deviations. Accidentally, the moderating effect of expressive suppression between emotional abuse and depressive symptom makes no sense. Figure 2 illustrates the moderating effect of expressive suppression.

**Table 3:** Result from PROCESS macro testing expressive suppression moderation model 1 and model 2.

**Effect^a^, Variable**	**R2**	**F**	**t**	**β**	**P**
Model 3 summary	0.2813	36.4894			<0.001
1. Effect of independent^b^ on dependent ^d^			11.5304	0.1723	<0.001
2. Effect of moderator^c^ on dependent^d^			7.1914	0.1835	<0.001
	R2	95%CI	t	β	P
3.Interaction effect^e^ on dependent ^d^	0.0044	0.0008, 0.0117	2.622	0.0063	0.0089
**Effect** ^a^ **, Variable**	**R2**	**F**	**t**	β	**P**
Model 4 summary	0.3165	43.1478			<0.001
1. Effect of independent^f^ on dependent^d^			13.7475	0.7243	<0.001
2. Effect of moderator^c^ on dependent^d^			6.653	0.1658	<0.001
	R2	95%CI	t	β	P
3.Interaction effect^g^ on dependent ^d^	0.0011	−0.0071, 0.0311	1.3402	0.112	0.1805

^a^Adjusted for age, gender, place of residence, resident student, parental absence, marital status of parents, only-child, teacher criticism, study stress.

^b^Childhood trauma.

^c^Expressive suppression.

^d^Depressive symptoms.

^e^Childhood trauma x expressive suppression.

^f^Emotional abuse.

^g^Emotional abuse x expressive suppression.

## Discussion

A noteworthy finding from our study was that childhood trauma, including neglect and abuse, emotion regulation, and depressive symptom, were all significantly associated among Zhuang teenagers who participated in this research. The score for childhood trauma was positively linked with the score for expressive suppression. Expressive suppression, that is, people occasionally control their emotions by preventing the behavioral expression of emotion after an emotional response has been generated (41); the depressive symptom score was positively correlated with the total childhood trauma score among Zhuang teenagers who participated in this study. A meta-analysis of 184 research revealed that childhood trauma is related to the incidence, onset, age, and duration of depressive symptom (10). Moreover, many individuals with dissociative depressive symptom reported early school dropout and sexual abuse in childhood (45). Our research revealed a correlation between a teenager's childhood trauma score and his or her depressive symptom score. Moreover, various childhood trauma both connect with depressive symptom. Moreover, a second meta-analysis revealed that distinct types of traumas in childhood have distinct consequences on depressive symptom (46). Since our study utilized the total childhood trauma score as the observation variable and simultaneously analyzed several types of traumas, the total childhood trauma score and emotion abuse score, respectively served as the observation variable. The cross-sectional study demonstrating a correlation between expressive suppression and depressive symptom in adults and adolescents (47) has often been interpreted as reflecting the impact of expressive suppression on depressive symptom. Concerning the effect of expressive suppression on depressive symptom, our study revealed a positive correlation between the expressive suppression score and the depressive symptom score. The current study provides more evidence in favor of our earlier research (48), suggesting a unidirectional association between depressive symptom and increased expressive suppression use.

Another result was that expressive suppression had to mediate and moderate effects on childhood trauma and depressive symptom. The findings demonstrated that childhood trauma positively predicted the Zhuang adolescents' expressive suppression and indirectly affected the Zhuang adolescents' depressive symptom through expressive suppression. However, for specific types of traumas, expressive suppression only had to mediate effects on emotional abuse and depressive symptom. Consistent with the hypothesis of ironic mental control processes (49), this study indicated that emotional maltreatment affected both depressive symptom and problem behaviors indirectly *via* expressive repression. In addition, the moderating effect demonstrated that expressive suppression amplified the effect of childhood trauma on the future development of depressive symptom (50–52). Consistent with earlier research, this study discovered that adolescents are particularly vulnerable to faulty emotion regulation, which may raise their risk for a psychiatric disorder (53). That is, expressive suppression, as a cognitive emotion regulation strategy, is harmful to human development (54). The passing of time cannot be reversed for adolescents but childhood traumas are irrevocable, although expressive repression can lessen their usage. For example, the preview study (55) indicated that expressive suppression had more depressive symptom, life satisfaction, greater self-esteem, and general well-being than cognitive reappraisal. Moreover, suppression of emotion suggests that the suppressed feeling continues to persist and build in an unresolved condition (21), and suppression efforts paradoxically make the target of suppression more accessible (49). This raises negative feelings but not behavioral issues, resulting in an increase in depressive symptom. According to these studies, individual differences in these two strategies may be linked to the development and maintenance of depressive disorders (56).

A study found an association between expressive suppression and unfavorable interpersonal outcomes, such as lower levels of liking, relationship satisfaction, and relationship quality (57). Through the adjustment of cognitive affective, individuals can improve their presentation and interpretation of events with the assistance of parental and peer support (58). As an essential factor influencing depressive symptom, expressive suppression can be practiced to limit its use with the assistance of family and friends, as well as cognitive intervention training. Therefore, reducing expressive suppression can be used as an intervention object and even as a treatment to lessen the risk of depressive symptom in Zhuang adolescents.

In summary, we investigated whether expressive suppression, as a cognitive emotion regulation approach, mediated and moderated the association between childhood trauma and increased risk of depressive symptom in a sample of Zhuang teenagers in China. Our findings validated prior findings that expressive suppression mediates and moderates the relationship between childhood trauma and depressive symptom. In addition, we elucidate the role of cognitive emotion regulation strategies in greater detail by examining various types of childhood trauma, and expressive suppression only had mediating effects on the connection between emotional abuse and higher risk of depressive symptom in this study.

## Limitations and strengths

This study is restricted by its cross-sectional methodology, which prohibited the identification of a causal association between childhood trauma and depressive symptom. Additionally, the sample was not necessarily representative of all Zhuang adolescents. Additionally, because the sample was single, mediating effects on the relationship between the other types of childhood traumas: emotional neglect, physical abuse and neglect, sexual abuse and depressive symptom did not appear. Future studies need further validation and discussion. We studied a sample of more than one thousand people, and the response rate was high; this was a strength of the study, therefore, the conclusions we found can be considered reliable. Our study confirmed that cognitive emotion regulation strategies attenuate modest relationships between childhood trauma and depressive symptom, providing a great empirical endorsement for previous research.

## Conclusions

This study's findings confirmed previous research indicating that the development of depressive symptom is mediated and moderated by the cognitive emotion management technique of expressive suppression. Inducing expressive repression can not only avoid depressive symptom among middle school adolescents but also enhance their future ability to overcome adversity. Although we demonstrate that cognitive emotion regulation strategies play a mediating and moderating role in the links between childhood trauma and depressive symptom, the mediating effects on the relationships between the other types of childhood traumas, including physical abuse and neglect, sexual abuse, emotional neglect, and depressive symptom, did not emerge.

## Data availability statement

The raw data supporting the conclusions of this article will be made available by the authors, without undue reservation.

## Author contributions

WY managed the literature search and analyses. YP, QW, and SZ designed the study and wrote the protocol. HH undertook the statistical analysis. LZ and WY wrote the first draft of the manuscript. QL, SP, and CD participated in data collection. JW participated in coaching as a leader. All authors contributed to and have approved the final manuscript.

## Funding

This research was supported by the Chinese National Nature Science Foundation (No. 81660569). The funders played no role in the study design, data collection and analysis, publication decision, or manuscript preparation.

## Conflict of interest

The authors declare that the research was conducted in the absence of any commercial or financial relationships that could be construed as a potential conflict of interest.

## Publisher's note

All claims expressed in this article are solely those of the authors and do not necessarily represent those of their affiliated organizations, or those of the publisher, the editors and the reviewers. Any product that may be evaluated in this article, or claim that may be made by its manufacturer, is not guaranteed or endorsed by the publisher.
